# Low Diagnostic Yield of Routine Cerebrospinal Fluid Analysis in Juvenile Stroke

**DOI:** 10.3389/fneur.2018.00694

**Published:** 2018-08-22

**Authors:** Alexandra Prakapenia, Kristian Barlinn, Lars-Peder Pallesen, Anne Köhler, Timo Siepmann, Simon Winzer, Jessica Barlinn, Dirk Daubner, Jennifer Linn, Heinz Reichmann, Volker Puetz

**Affiliations:** ^1^Department of Neurology, Carl Gustav Carus University Hospital, Technische Universität Dresden, Dresden, Germany; ^2^Institute of Neuroradiology, Carl Gustav Carus University Hospital, Technische Universität Dresden, Dresden, Germany

**Keywords:** juvenile stroke, cerebrospinal fluid analysis, vasculitis, etiology, secondary stroke prevention

## Abstract

**Background:** The diagnostic value of cerebrospinal fluid (CSF) analysis in juvenile stroke, i.e., stroke in young adult patients, is not well studied. We sought to determine the therapeutic impact of routine CSF-analysis in young adults with acute ischemic stroke or transient ischemic attack (TIA).

**Methods:** We abstracted data from patients with acute cerebral ischemia aged 18–45 years who were consecutively admitted to our stroke center between 01/2008 and 12/2015. We routinely performed CSF-analysis in patients with hitherto unknown stroke etiology after complete diagnostic work up. We assessed the frequency and underlying causes of abnormal CSF-findings and their impact on secondary stroke prevention therapy.

**Results:** Among 379 patients (median [IQR:IQR3-IQR1] age 39 [10:43-33] years, 48% female) with acute ischemic stroke (*n* = 306) or TIA (*n* = 73), CSF analysis was performed in 201 patients (53%). Of these, 25 patients (12.4 %) had CSF pleocytosis (leucocyte cell count ≥ 5 Mpt/L), that was rated as non-specific (e.g., traumatic lumbar puncture, reactive pleocytosis) in 22 patients. Only 3 patients (1.5% of all patients who underwent CSF-analysis) with CSF-pleocytosis had specific CSF-findings that were related to stroke etiology and affected secondary stroke prevention therapy. Imaging findings had already suggested cerebral vasculitis in two of these patients.

**Conclusions:** The diagnostic yield of routine CSF-analysis in juvenile stroke was remarkably low in our study. Our data suggest that CSF-analysis should only be performed if further findings raise the suspicion of cerebral vasculitis.

## Introduction

Acute ischemic stroke is the second most frequent cause of death and most frequent cause of acquired adult disability worldwide if all adult age subgroups are considered (1). However, juvenile stroke, i.e., stroke in a patient aged 18 to 45 years is rather infrequent and is characterized by improved functional outcome and survival rates. (2, 3) Due to the reduced prevalence of classical vascular risk factors and common stroke causes, the detection of stroke etiology is more difficult in these patients (4–6).

Compared with ischemic stroke in older patients, stroke of other determined etiology (e.g., dissection, Fabry's disease, central nervous system [CNS] vasculitis) and stroke of undetermined etiology according to Trial of ORG 10172 in Acute Stroke Treatment (TOAST) criteria are more frequent in juvenile stroke. (7) With the intention not to miss uncommon causes, etiologic work-up is frequently extensive including invasive procedures like transesophageal echocardiography (TEE), digital subtraction angiography (DSA), and cerebrospinal fluid (CSF) analysis by means of diagnostic lumbar puncture. (6) However, the diagnostic value of CSF-analysis in the identification of stroke etiology in juvenile stroke is not well studied.

Current stroke guidelines do not provide specific guidance for the identification of young stroke patients in whom CSF-analysis should be performed as part of the diagnostic work-up (8). Whereas some smaller studies have suggested that CSF-analysis should routinely be performed in young stroke patients (9, 10), the additional diagnostic value of CSF-analysis for the identification of stroke etiology is thought to be low (9).

The aim of our study was to analyze the frequency of abnormal CSF-findings and to determine the diagnostic and therapeutic impact of routine CSF-analysis in young adult patients with acute ischemic stroke or transient ischemic attack (TIA).

## Materials and methods

We performed a retrospective cohort study of consecutive young adult patients with acute ischemic stroke or TIA who presented to our tertiary care hospital between January 2008 and December 2015. Data collection was performed via review of the electronic-hospital patient information system (Orbis, AGFA-HealthCare, Bonn, Germany) including discharge summaries and documentation of clinical, laboratory and imaging data. Inclusion criteria were patient age between 18 and 45 years and discharge diagnosis of acute ischemic stroke or TIA according to International Statistical Classification of Diseases and Related Health Problems (ICD)-10 codes (I63, I64, and G45).

Routine work-up included brain imaging with computed tomography (CT) or magnetic resonance imaging (MRI), Duplex-ultrasound of extracranial and intracranial arteries, cardiac work-up including Holter-ECG for at least 24 h, transthoracic and transesophageal echocardiography, serological screening for coagulopathies and Fabry's disease and systemic vasculitis antibody panel. Routine systemic vasculitis antibody panel included antineutrophil cytoplasmic-[ANCA], anti-nuclear-[ANA], extractable nuclear antigens-[ENA-A], cardiolipin- and anti-double stranded DNA antibodies [Anti-dsDNA]). We recorded vascular risk factors and medical conditions of interest including arterial hypertension, lipid disorders, sleep apnea, migraine, illicit drug abuse, defined as patients‘ statements about regular consumption of non-legal drugs (e.g., cocaine, marihuana, heroin, crystal-meth), diabetes mellitus, smoking, coronary artery disease, oral contraception, positive family history, and—history of prior ischemic stroke or TIA. We also collected data on acute treatment, including intravenous thrombolysis and endovascular thrombectomy, stroke etiology by TOAST criteria as judged by the treating stroke neurologist and the specific stroke prevention therapy at discharge. We collected data on baseline stroke severity as measured with the National Institutes of Health Stroke Scale (NIHSS) score and functional status at discharge as determined with the modified Rankin Scale (mRS) score. We defined favorable functional outcome at discharge as mRS scores of 0–2.

All results from CSF analysis, serology, microbiological, and virological studies were interpreted according to internal normal values at our institution.

Our local institutional ethics committee Technical University of Dresden approved the conduction of this study (EK 511122016).

### Cerebrospinal fluid analysis

Per institutional routine during the study period, we performed a diagnostic lumbar puncture in young adults with ischemic stroke or TIA with hitherto unknown stroke etiology and in patients with findings on routine work-up suggesting cerebral vasculitis. The final decision to perform a diagnostic lumbar puncture was at the discretion of the treating stroke neurologist. All patients or their legal representatives signed informed consent prior to lumbar puncture. In case of CSF-pleocytosis (CSF cell count ≥ 5Mpt/L), we assessed for virological and microbiological abnormalities. The routine CSF-analysis in these patients consisted of tests for Lyme's disease, neurosyphilis, herpes simplex virus, and varicella zoster virus polymerase chain reaction (PCR), microbiological culture and tests for HIV infection. We rated CSF-pleocytosis as non-specific if the results of these tests were normal and if vascular imaging and vasculitis antibody panel did not suggest cerebral or systemic vasculitis. The CSF-pleocytosis in these patients was then rated to be due to CSF-probe taken from an external ventricular drainage (EVD), as reactive pleocytosis due to an acute ischemic stroke, or as result of traumatic lumbar puncture. In doubt, CSF-analysis was repeated. The final interpretation of CSF-pleocytosis was based on the discretion of the treating stroke neurologist.

### Imaging

We collected data about the type of imaging performed, the presence and localization of acute or subacute ischemic changes and the vascular status based on CT angiography (CTA) or MR angiography (MRA) results as stated in the final neuroradiology reports. All imaging findings suspicious of cerebral vasculitis were documented. For diagnosis of arterial dissection, we routinely performed dissection sensitive MRI sequences (T1-weighted imaging with fat-saturation) (11, 12, 13).

### Statistical analysis

Statistical analyses were performed with STATA software (version 12.1, StataCorp. College Station, Tx). Continuous variables are presented as mean ± standard deviation (SD) for normally distributed or as median (interquartile range, IQR: IQR3-IQR1) for skewed distributed data, whereas non-continuous variables are presented as percentages. Statistical comparisons were performed using Chi-square test, Fisher's exact test, Student's test and Wilcoxon rank sum, where appropriate. Univariate and multivariable analyses were conducted to evaluate associations among patients with and without CSF analysis controlling for age, gender, vascular risk factors, and further clinical variables. A *p*-value of < 0.05 was considered to be statistically significant.

## Results

### Patients

**Figure 1:**
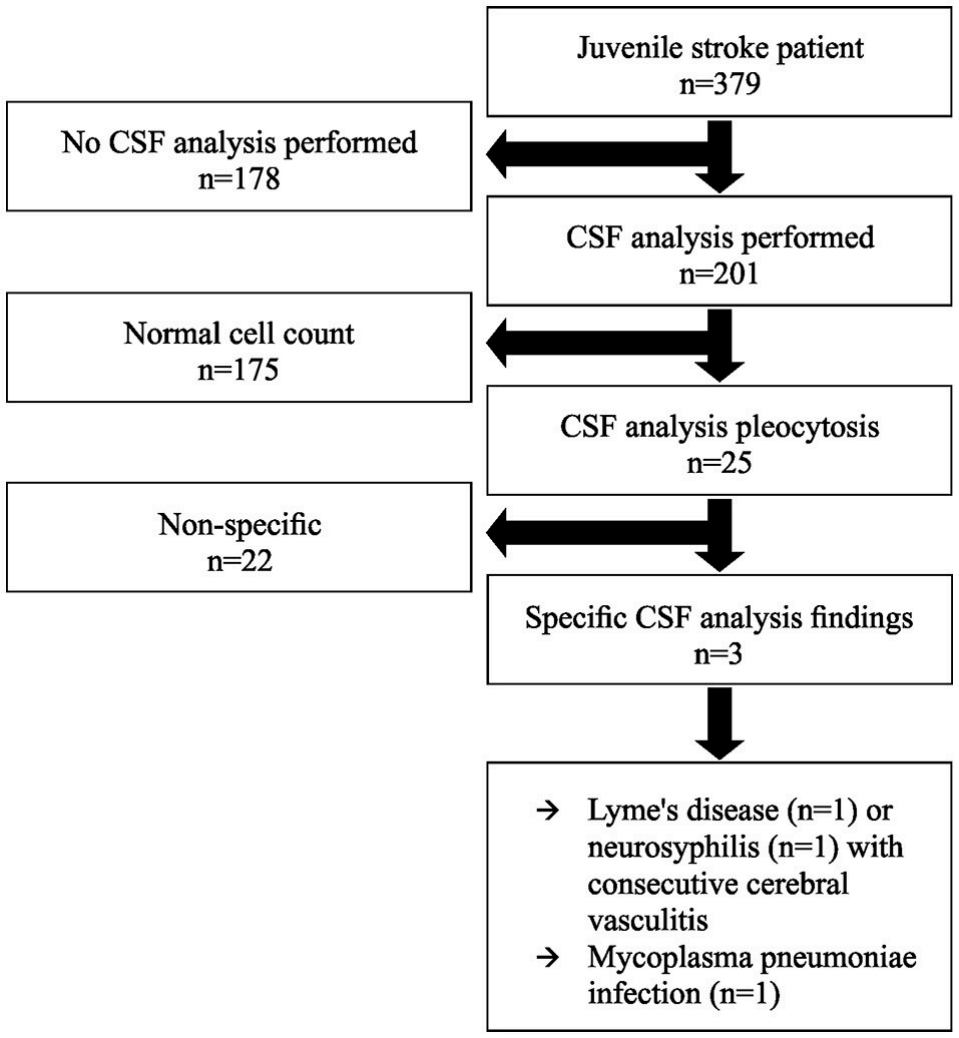
Study flow chart. CSF indicates cerebrospinal fluid.

During the study period, 379 patients aged between 18 and 45 years were admitted to our tertiary care hospital with acute ischemic stroke (*n* = 306) or TIA (*n* = 73). The median (interquartile range, IQR [IQR3-IQR1]) age was 39 (10 [43-33]) years and 48% were female. The median (IQR [IQR3-IQR1]) NIHSS score on admission was 2 (6 [5.5–0]) points. We performed a cerebrospinal fluid analysis for etiological work-up in 201 patients (53%). The study flow chart is depicted in Figure 1.

Compared to patients without CSF-analysis, patients who underwent CSF-analysis were younger, more frequently female, were more frequently treated with endovascular therapy (EVT), more commonly suffered from coronary heart disease and more frequently had a positive family history for stroke. Baseline characteristics are summarized in Table 1.

**Table 1:** Baseline characteristics of patients with and without CSF analysis.

**Variable**	**CSF-analysis (*n* = 201)**	**No CSF- analysis (*n* = 178)**	***p*-value**


Age, years (IQR; IQR3-IQR1)	38 (11; 42–31)	41 (9; 44–35)	0.001
Female, n (%)	107 (53)	75 (42)	0.031
Ischemic stroke, n (%)	168 (84)	138 (78)	0.136
TIA, n (%)	33 (16)	40 (22)	0.136
Baseline NIHSS score, median (IQR; IQR3-IQR1)	1 (4; 4–0)	2 (8; 8–0)	0.115
IVT, n (%)	32 (16)	35 (20)	0.340
EVT, n (%)	8 (4)	17 (10)	0.029
**VASCULAR RISK FACTORS AND MEDICAL CONDITIONS**			
Arterial hypertension, *n* (%)	37 (18)	39 (22)	0.395
Lipid disorders, *n* (%)	10 (5)	16 (9)	0.123
Smoking, *n* (%)	79 (39)	77 (43)	0.435
Sleep apnea, *n* (%)	3 (2)	3 (2)	1.00
Migraine, *n* (%)	16 (8)	7 (4)	0.101
Illicit drug abuse, *n* (%)	8 (4)	5 (3)	0.584
Diabetes mellitus, *n* (%)	3 (2)	9 (5)	0.075
Coronary artery disease, *n* (%)	0 (0)	5 (3)	0.022
Oral contraception, *n* (%)	28 (14)	8 (5)	0.002
Positive family history, *n* (%)	29 (14)	13 (7)	0.33
Previous ischemic stroke or TIA, *n* (%)	14 (7)	21 (12)	0.105

*IQR(IQR3-IQR1) indicates interquartile range; TIA, transient ischemic attack; NIHSS, National Institute of Health Stroke Scale; mRS, modified Rankin Scale; IVT, intravenous thrombolysis; EVT, endovascular thrombectomy*.

Overall, at discharge, 292 patients (77 %) had a favorable functional outcome, 76 patients (20 %) had an unfavorable functional outcome and 11 patients (3 %) were deceased. Compared to patients who did not receive a CSF-analysis, patients who underwent a CSF-analysis more frequently had a favorable functional outcome at discharge (69 vs. 84% respectively; *p* = 0.001).

### Stroke etiology

The distribution of stroke etiology by TOAST criteria is presented in Table 2. The stroke etiology in patients who received a CSF-analysis was more often unknown compared to patients who did not receive a CSF-analysis. Other determined etiologies were more frequently detected in patients who did not receive a CSF-analysis. None of 176 patients who were screened for Fabry's disease had a positive test result.

**Table 2:** Etiology of stroke as determined by TOAST-criteria of patients with CSF- analysis compared to patients without CSF-analysis.

**Variable**	**CSF-analysis (*n* = 201)**	**No CSF-analysis (*n* = 178)**	***p*-value**
**ETIOLOGY**, ***N*** **(%)**			
Macroangiopathy	10 (5)	13 (7)	< 0.0001
Cardioembolic	21 (10)	19 (11)	
Small vessel disease	4 (2)	7 (4)	
Unknown etiology	139 (69)	80 (45)	
Other determined etiology, *n* (%):	22 (11)	58 (33)	
•Cerebral vasculitis	4 (2)	3 (2)	
•Dissection	7 (3)	46 (26)	
•Hypercoagulable state	8 (4)	3 (2)	
•CADASIL	1 (1)	0	
•Moya-Moya-disease	1 (1)	5 (3)	
•FMD	1 (1)	0	
•MELAS	0	1 (1)	

*TOAST indicates “Trial of Org 10172 in Acute Stroke Treatment”; CADASIL, Cerebral Autosomal Dominant Arteriopathy with Subcortical Infarcts and Leukoencephalopathy; MELAS, mitochondrial encephalomyopathy with lactic acidosis and stroke-like episodes; FMD, fibromuscular dysplasia*.

Among 379 patients analyzed in this study, 7 young stroke patients have a diagnosis of primary CNS-vasculitis prior to their hospital stay. CSF-analysis war performed in 4 of these patients, but none of them exhibited CSF- pleocytosis.

### Results of CSF-analysis

Among the 201 patients who received a CSF-analysis, 25 patients (12.4%) had CSF-pleocytosis with a median cell count of 13 Mpt/L (Range 6–73; Table 3). Of these, 3 patients (1.5% of all patients, who underwent CSF analysis) had specific CSF-abnormalities that were thought to be related to stroke etiology (Figure 1). Among the remaining 22 patients with CSF-pleocytosis, we rated abnormal CSF-results as non-specific. All microbiological, viral, and immunological analyses were normal in these patients. The final clinical interpretation of CSF-pleocytosis in these patients was traumatic lumbar puncture (*n* = 8), reactive pleocytosis due to acute stroke (*n* = 13), or as CSF-specimen was taken from an external ventricular drainage (*n* = 2).

**Table 3:** Baseline characteristics of CSF-analysis of patients with pleocytosis.

**Patient, number**	**Age, years (range)**	**ischemic event**	**Baseline NIHSS score**	**Cell count/Follow up, Mpt/L**	**Protein level, mg/l**	**Glucose, mmol/l**	**Lactate, mmol/l**	**Description (cause of pleocytosis)**
1	41–45	Stroke	0	73/53	3000	2	2	Lyme's disease
2	41–45	Stroke	2	64	1282	4	2	non-specific (traumatic)
3	26–30	Stroke	5	52	537	3	2	non-specific (EVD)
4	36–40	Stroke	3	43/19	452	4	2	non-specific (reactive)
5	41–45	Stroke	3	40/3	580	4	2	non-specific (reactive)
6	41–45	Stroke	13	38/8	436	5	2	non-specific (EVD)
7	26–30	TIA	1	25/32/7	447	2	3	Mycoplasma pneumoniae
8	18–25	Stroke	5	29	313	3	1	non-specific (reactive)
9	36–40	Stroke	15	27	270	3	2	non-specific (reactive)
10	36–40	Stroke	5	18	540	4	2	non-specific (reactive)
11	26–30	Stroke	32	16/22	2166	4	3	neurosyphilis
12	36–40	Stroke	1	15	585	3	2	non-specific (traumatic)
13	36–40	Stroke	2	11	1507	3	2	non-specific (traumatic)
14	41–45	Stroke	1	11	423	3	3	non-specific (reactive)
15	18–25	Stroke	7	8	445	3	2	non-specific (reactive)
16	41–45	TIA	9	8	309	3	2	non-specific (reactive)
17	36–40	TIA	3	7	1212	4	2	non-specific (reactive)
18	36–40	Stroke	4	7	395	4	1	non-specific (reactive)
19	31–35	Stroke	1	7	360	4	2	non-specific (reactive)
20	31–35	Stroke	7	7	800	4	2	non-specific (reactive)
21	31–35	Stroke	2	6	272	4	2	non-specific (traumatic)
22	41–45	Stroke	2	6	594	4	2	non-specific (reactive)
23	36–40	Stroke	13	6	348	4	2	non-specific (traumatic)
24	18–25	TIA	0	6	312	3	1	non-specific (traumatic)
25	41–45	Stroke	0	6	433	3	2	non-specific (reactive)

*EVD indicates external ventricular drainage*.

### Characteristics of patients with specific CSF-findings

The results of the three patients with CSF-pleocytosis and specific findings that were thought to be related to stroke etiology are summarized below.

**Figure 2:**
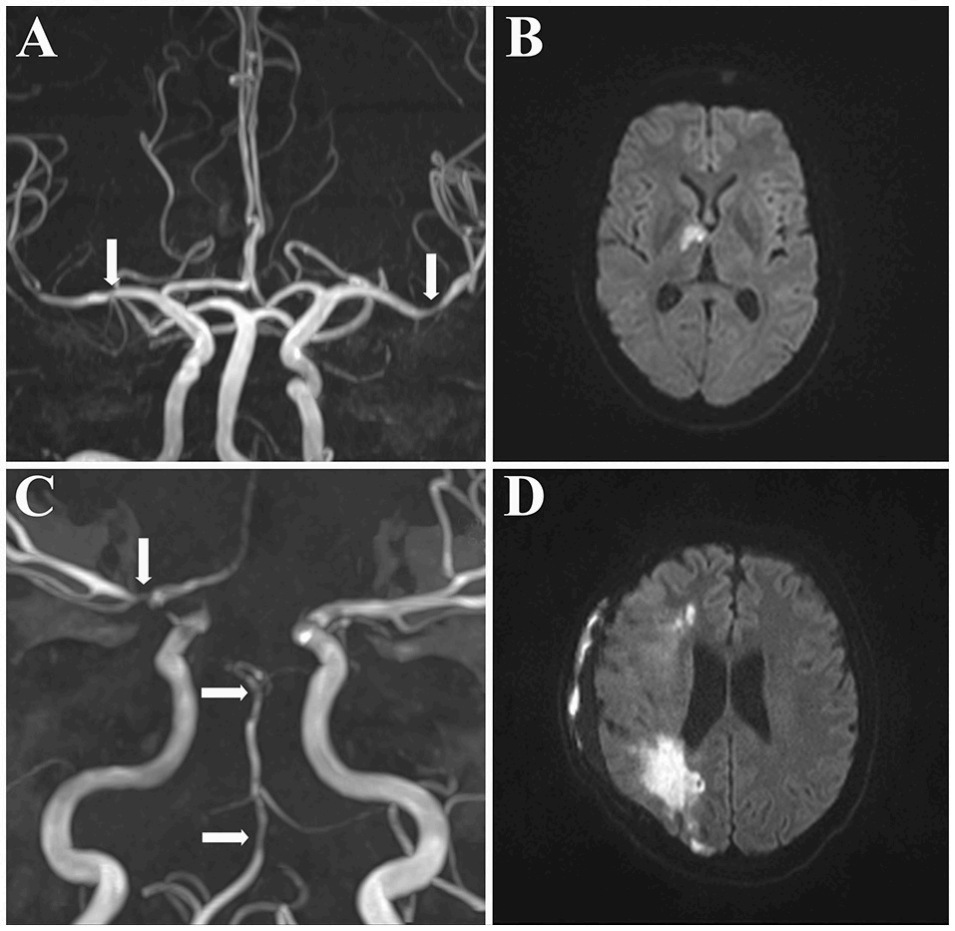
MR angiography of 2 patients with cerebral vasculitis. MR angiography with multiple vessel stenoses suggesting cerebral vasculitis. Both patients had CSF-pleocytosis (cell count 73 Mpt/L and 16 Mpt/L, respectively) and were diagnosed to have neuroborreliosis **(A,B)** or neurosyphilis **(C,D)**. Arrows indicate caliber irregularities of the right middle cerebral artery **(A)** or the basilar artery **(C)** on TOF-MRA. **(B,D)** indicate corresponding DWI-lesions. TOF-MRA indicates Time-of-Flight magnetic resonance angiography; DWI, diffusion weighted imaging; MR, magnetic resonance.

**Patient 1** (number 1 in Table 3): This patient had CSF-pleocytosis of 73 Mpt/L and elevated CSF-protein of 3,000 mg/l. Calculated antibiotic therapy with ceftriaxone and antiviral therapy with aciclovir were initiated. Time-of-flight (TOF) MR angiography had already demonstrated irregularities of vessel calibers (Figure 2A) corresponding to acute diffusion weighted imaging (DWI) lesions suggestive of cerebral vasculitis (Figure 2B). Specific intrathecal borrelia burgorferi antibodies established the diagnosis of Lyme's disease and antiviral therapy was discontinued. Control CSF-analysis after 1 week showed a decreasing cell count (53Mp/l). Antibiotic therapy with ceftriaxone was continued for 3 weeks. After 3 months the patient had no neurological deficit (mRS score 1).

**Patient 2** (number 11 in Table 3): This patient had CSF-pleocytosis of 16 Mpt/L and increased CSF-protein level of 2,166 mg/l. Calculated antibiotic therapy with ceftriaxone was initiated. MRI including TOF-MRA had demonstrated multiple vessel stenoses (Figure 2C) with corresponding DWI-lesions (Figure 2D). Microbiological assessments established the diagnosis of neurosyphilis and antibiotic therapy with ceftriaxone was continued for 3 weeks. This patient was further found to be HIV positive and highly active antiretroviral therapy (HAART) was initiated. Despite antibiotic and antiretroviral therapy, this patient worsened clinically and suffered malignant middle cerebral artery infarction requiring decompressive craniectomy. His discharge mRS score was 3.

**Patient 3** (number 7 in Table 3): This patient had CSF-pleocytosis of 25 Mpt/L but otherwise normal CSF-results. Serological work-up demonstrated mycoplasma pneumonia infection with positive serum mycoplasma pneumoniae IgM and IgA antibodies, which was rated as a contributing for stroke pathophysiology (14, 15). Due to lack of data, no specific therapy was initiated initially and the patient was treated with aspirin. Four months later, this patient represented with recurrent TIA. Repeated lumbar puncture revealed persistent CSF-pleocytosis (32 Mpt/L). Antibiotic therapy with clarithromycin was now initiated and continued for 2 weeks. Her discharge mRS score was 0. In follow-up CSF-analysis after 6 months without further cerebrovascular events, there was near normalization of CSF-pleocytosis (7 Mpt/L) and then negative serum value for mycoplasma pneumoniae IgA antibodies and borderline value for IgM antibodies.

## Discussion

Our study among a large cohort of young (18–45 years) adults with acute ischemic stroke or TIA demonstrates a low diagnostic yield of routine CSF-analysis to determine the etiology of acute cerebral ischemia. Only 1.5% of all patients who underwent a diagnostic lumbar puncture in our study had specific CSF-findings that were thought to be related to stroke etiology. Consequently, the therapeutic impact of CSF-analysis was low and confined to three patients who received specific antibiotic therapy as a consequence of CSF-results. Moreover, imaging findings had already suggested cerebral vasculitis in two of these patients. According to current guidelines, there are no specific recommendations for the identification of stroke etiology in young adults with acute ischemic stroke or TIA (8). Most studies that have analyzed the diagnostic relevance of CSF-analysis in juvenile stroke are based of small patient cohorts or case reports (9, 16, 17). The results of our study are in line with a previous report on stroke patients aged below 55 years with a total of 32 CSF-analyses performed (9). In this study, relevant information regarding stroke etiology was described in one patient only. In the context with our results, these findings suggest that CSF-analysis should only be performed at the end of routine diagnostic work up of juvenile stroke patients with hitherto unknown stroke etiology or if clinical symptoms or imaging findings are suggestive of cerebral vasculitis, infection or immunodeficiency, respectively (8, 18–20).

Due to the lower prevalence of classical vascular risk factors, the etiology of juvenile stroke has a different focus compared to stroke etiology in older patients. Besides coagulation, disorders, CNS vasculitis, dissection, and genetic disorders such as Fabry's disease are increasingly important (21, 22). However, no Fabry's disease was detected among 176 patients who received specific tests in our study and apart from dissection, other specific stroke etiologies were rarely identified in our cohort.

Stroke etiology was thought to be secondary to mycoplasma pneumoniae infection in one patient in our study who had CSF-pleocytosis. An association of mycoplasma pneumoniae infection with ischemic stroke particularly in children and young adults has been described in literature (15, 23, 24). While the exact pathomechanism is unknown, a correlation between thromboembolism and cell inflammation has been suggested (14, 15). No standardized treatment regimen besides symptomatic therapy has been suggested in these studies. Our patient had recurrent TIA prior to initiation of antibiotic therapy but suffered no further event after antibiotic therapy with clarithromycin and CSF-pleocytosis subsided. It needs to be determined whether such antibiotic therapy has an impact on stroke recurrence risk in future studies.

Our study has limitations. First, the study design was a monocentric retrospective study. Our data may not be generalizable to other regions with different socioeconomic background where stroke etiologies in young adults may vary broadly. Second, the decision to perform a lumbar puncture was based on the discretion of the treating stroke neurologist causing a potential for selection bias. In fact, some patients with unknown etiology according to TOAST criteria received no CSF-analysis whereas some patients who received CSF-analysis had other specific stroke etiologies. Third, CSF-pleocytosis was thought to be non-specific based on the results of microbiological and virological tests in the majority of patients in our study. We cannot exclude that CSF-pleocytosis may have had pathophysiological relevance in some of these patients. As VZV vasculitis was only excluded by means of PCR results and not based on titer-increase in repeated serological studies. We cannot exclude that VZV infection was not accurately detected in some patients. Moreover, a significant number of patients had TIA rather than ischemic stroke. We cannot exclude that some of these patients may have had non-stroke diagnoses like headache with neurological deficits and lymphocytosis (HaNDL) mimicking ischemic stroke (25).

In summary, the diagnostic yield of routine CSF-analysis in young adults aged 18–45 years with acute ischemic stroke or TIA was remarkably low in our study. Our data suggest that CSF-analysis should only be performed if further clinical or imaging findings are suspicious for cerebral vasculitis in patients with hitherto unknown stroke etiology.

## Data availability statements

The datasets for this manuscript are not publicly available for data protection reasons. Requests to access the datasets should be directed to Alexandra Prakapenia, alexandra.prakapenia@ukdd.de.

## Author contributions

AP acquired and interpreted the data, coordinated the study and drafted the manuscript. KB interpreted the data, performed the statistical analysis, and reviewed the manuscript for content. L-PP, AK, TS, and JB revised the manuscript for content. HR supervised the study and revised the manuscript for content. SW created figures und reviewed the manuscript for content. DD and JL acquired imaging data and reviewed the manuscript for content. VP designed und coordinated the study and reviewed the manuscript for content.

### Conflict of interest statement

The authors declare that the research was conducted in the absence of any commercial or financial relationships that could be construed as a potential conflict of interest.
